# The Era of Antimicrobial Peptides: Use of Hepcidins to Prevent or Treat Bacterial Infections and Iron Disorders

**DOI:** 10.3389/fimmu.2021.754437

**Published:** 2021-09-27

**Authors:** Carolina Barroso, Pedro Carvalho, Magda Nunes, José F. M. Gonçalves, Pedro N. S. Rodrigues, João V. Neves

**Affiliations:** ^1^ i3S - Instituto de Investigação e Inovação em Saúde, Universidade do Porto, Porto, Portugal; ^2^ Iron and Innate Immunity, IBMC – Instituto de Biologia Molecular e Celular, Universidade do Porto, Porto, Portugal; ^3^ Programa Doutoral em Biologia Molecular e Celular (MCbiology), ICBAS - Instituto de Ciências Biomédicas Abel Salazar, Universidade do Porto, Porto, Portugal; ^4^ ICBAS - Instituto de Ciências Biomédicas Abel Salazar, Universidade do Porto, Porto, Portugal

**Keywords:** antimicrobial peptides, hepcidin, infection, iron homeostasis, aquaculture, European sea bass (*Dicentrarchus labrax*)

## Abstract

The current treatments applied in aquaculture to limit disease dissemination are mostly based on the use of antibiotics, either as prophylactic or therapeutic agents, with vaccines being available for a limited number of fish species and pathogens. Antimicrobial peptides are considered as promising novel substances to be used in aquaculture, due to their antimicrobial and immunomodulatory activities. Hepcidin, the major iron metabolism regulator, is found as a single gene in most mammals, but in certain fish species, including the European sea bass (*Dicentrarchus labrax*), two different hepcidin types are found, with specialized roles: the single type 1 hepcidin is involved in iron homeostasis trough the regulation of ferroportin, the only known iron exporter; and the various type 2 hepcidins present antimicrobial activity against a number of different pathogens. In this study, we tested the administration of sea bass derived hepcidins in models of infection and iron overload. Administration with hamp2 substantially reduced fish mortalities and bacterial loads, presenting itself as a viable alternative to the use of antibiotics. On the other hand, hamp1 seems to attenuate the effects of iron overload. Further studies are necessary to test the potential protective effects of hamp2 against other pathogens, as well as to understand how hamp2 stimulate the inflammatory responses, leading to an increased fish survival upon infection.

## Introduction

During the last decades, aquaculture became the fastest growing food production sector, with nearly half of fishes consumed worldwide being raised on fish farms (1). However, fish species are produced under intensive aquaculture practices, leading to the appearance of disease outbreaks, mostly caused by bacteria or viruses (2), associated with high mortalities and production losses (3). Vaccination to prevent disease in aquaculture is being routinely used in certain fish species, mostly salmonids, but efficient vaccines for other fishes and pathogens are still lacking. As such, fish farmers rely on the use of antibiotics, for prophylactic and therapeutic purposes (4, 5). However, the misuse of antibiotics in animal production, which led to the emergence of antibiotic-resistant microorganisms, with serious public health implications, as well as the inability of antibiotics in treating viral diseases, urgently presses for the development of alternatives to these drugs (4, 6). Antimicrobial peptides (AMPs) are considered as promising novel compounds to be used in aquaculture industry, due to their antimicrobial properties, immunomodulatory roles and reduced probability to develop bacterial resistance (7–9). Fish present an extraordinary repertoire of AMPs, including the major groups of peptides, such as hepcidins, beta-defensins, cathelicidins and the fish specific piscidins (10, 11).

Hepcidin is a small cysteine rich peptide, first described in mammals by Krause et al. and named LEAP-1 (liver-expressed antimicrobial peptide) (12). Later, Park et al. isolated the same peptide and named hepcidin due to its hepatic expression and bacterial killing *in vitro* (13). However, the major role of hepcidin is the regulation of iron metabolism, by inhibiting post-translationally the iron exporter ferroportin (14, 15). Hepcidin is induced by iron overload and infection or inflammation and inhibited by iron deficiency and hypoxia (16, 17). During an inflammatory stimulus, hepcidin is induced by inflammatory cytokines, leading to a decrease of iron release from hepatocytes, macrophages and enterocytes, trough ferroportin internalization and degradation. As a consequence, circulating iron is limited, as well as its availability for pathogens. However, as a long term effect, this also limits iron availability for erythropoiesis, leading to a condition known as anemia of inflammation or anemia of chronic disease (16, 18).

Although most mammals present a single hepcidin [with the mouse being an exception (19, 20)], with a dual function as an iron regulator and antimicrobial molecule, genome duplications and positive selection led to the appearance of multiple copies of hepcidin in certain fish species (21–25). In the European sea bass (*Dicentrarchus labrax*), two different hepcidin types were described, with specialized roles: the single type 1 hepcidin (hamp1) is homologous to the mammalian counterpart, with a preponderant role on iron metabolism; and the various type 2 hepcidins (hamp2) show a direct activity against different bacteria (26). Teleost fish presenting two hepcidin types show a considerable degree of subfunctionalization, with hamp1 having a conserved inhibitory function on ferroportin, while the multiple hamp2 mostly performs antimicrobial roles (26, 27). Thus, while the antimicrobial role of hepcidin in mammals is limited, the presence of several type 2 hepcidins in some fish species indicates a more significant role of hepcidin as an antimicrobial molecule in fish (26).

Several studies have shown the activity of hepcidin against an array of pathogens *in vitro* (28–32). *In vivo*, treatment with hepcidin resulted in an increased fish survival and reduced bacterial or viral loads (28, 33–35). In sea bass, only one report addressed the effects of hepcidin administration in infected fish with *Vibrio anguillarum*, with sea bass presenting a higher resistance and reduced mortalities (36). These studies show the potential of hepcidin to be used as an alternative to the antimicrobial treatments currently applied in aquaculture. However, information concerning the effects of hepcidin administration in fish is still very scarce, particularly the use of hepcidin type 1 in models of iron disorders.

In this study, we tested the administration of hamp1 or hamp2 in our experimental models of infection with *Photobacterium damselae* spp. *piscicida* and iron overload. Our results demonstrate a clear beneficial effect of hamp2 in infected animals, as this molecule is capable of controlling bacterial infections and reducing fish mortalities, without interfering with iron metabolism. On the other hand, hamp1 administration seems to attenuate the effects of experimental iron overload. As such, fish hepcidins can be differentially applied in the treatment or prevention of infections and iron disorders. Further studies are necessary to test the potential protective effect of hamp2 against other pathogens, as well as to understand how hamp2 stimulate fish immune responses, leading to a higher fish resistance to infection.

## Methods

### Animals

Healthy European sea bass (*Dicentrarchus labrax*), with an average weight of 50 g, were provided by a commercial fish farm in the north of Spain (Sonríonansa S.L., Pesués, Cantabria, Spain). Prior to the experiments, fish were acclimated for 30 days to the fish holding facilities of the Instituto de Ciências Biomédicas Abel Salazar (ICBAS), Porto. Fish were kept in 110 liters recirculating sea water (28 ± 1 ‰ salinity) tanks at 22 ± 1°C, with a 13/11-hour light/dark cycle and fed daily *ad libitum* with commercial fish feed with an iron content of approximately 200 mg iron/kg feed. Before each treatment, fish were anaesthetized with ethylene glycol monophenyl ether (2-phenoxyethanol, 3 mL/10 L, Merck, Algés, Portugal). All animal experiments were carried out in strict compliance with national and international animal use ethics guidelines (including ARRIVE guidelines), approved by the animal welfare and ethic committees of ICBAS (permit P293/2019/ORBEA, 05/04/2019) and conducted by experienced and trained FELASA Function A+B+D investigators.

### *In Vivo* Experimental Models

**Figure 1:**
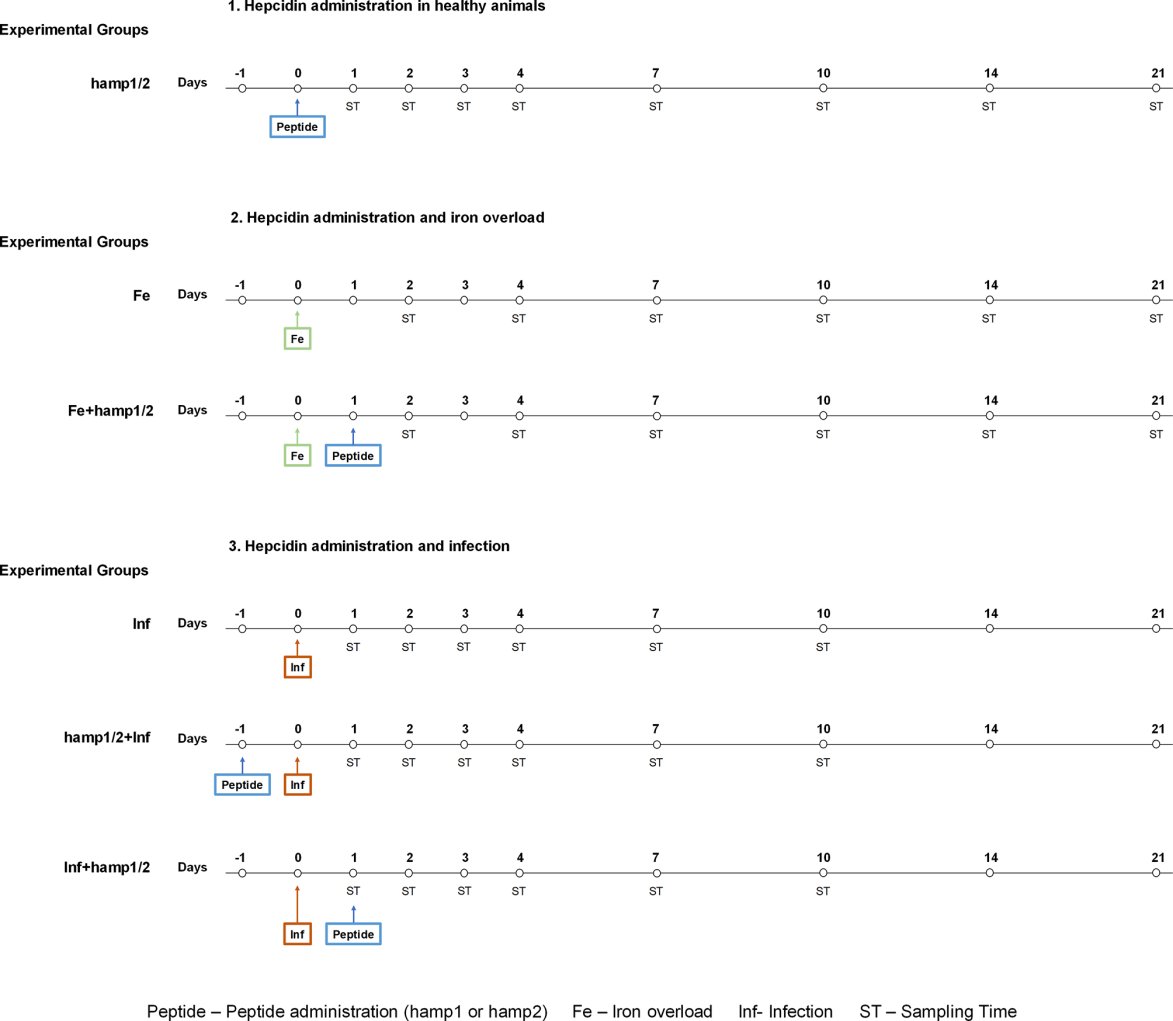
Experimental design. Healthy sea bass were first treated with hamp1 or hamp2. Then, different models were established: Iron overload (5mg of iron dextran/fish); iron overload followed by hamp1 or hamp2 administration, 24 hours later; Infection (10^5^ CFU of *P. damselae* strain PP3/fish); Pre-treatment with hamp1 or hamp2 followed by infection with PP3, 24 hours later; Post-treatment with hamp1 or hamp2, 24 hours after infection with PP3. Samples from fish were collected after 1, 2, 3, 4, 7, 10, 14 and 21 days of experiment.

To evaluate the effects of hamp1 or hamp2 administration in different conditions, several experimental models were established (**Figure 1**). Five fish from each experimental group were collected at the various time points after treatments and euthanized with an overdose of anesthetic. Blood and serum were collected for hematological and serological parameters determination. Animals were then dissected and tissues excised and snap frozen in liquid nitrogen and stored at -80°C, for further use in tissue iron content determination and gene expression. Mortality was assessed during the experimental infections. Colony forming units (CFU) counts in the spleen and head kidney of infected fish were performed. Briefly, spleen and head kidney were aseptically collected, homogenized in TSB 1% NaCl, serially diluted, plated on TSA 1% NaCl and incubated at 25°C for 24-48 hours. No animals were excluded in any of the experiments.

#### Peptide Administration

Fish were intraperitoneally injected with commercially synthesized sea bass hepcidin peptides, either with 100 µl of a 50 µM solution of hamp1 (QSHLSLCRWCCNCCRGNKGCGFCCKF), or hamp2 (HSSPGGCRFCCNCCPNMSGCGVCCRF) (Bachem AG, Bubendorf, Switzerland), diluted in sterile PBS. Samples were collected after 1, 2, 3, 4, 7, 10, 14, and 21 days post-peptide administration.

#### Iron Overload

To induce iron overload, fish were intraperitoneally injected with 100 µl iron dextran (5 mg) (Sigma-Aldrich, St. Louis MO, USA) diluted in sterile PBS to a final concentration of 50 mg/ml. Samples were collected after 2, 4, 7, 10, 14, and 21 days post-iron administration.

#### Iron Overload and Peptide Administration

Fish were first injected with iron dextran, followed by administration of either hamp1 or hamp2 peptides 24 hours later, as previously described. Samples were collected after 2, 4, 7, 10, 14, and 21 days post-iron administration.

#### Infection

*Photobacterium damselae spp piscicida* strain PP3 was cultured to midlogarithmic growth in tryptic soy broth growth medium, supplemented with 1% NaCl. After measuring absorbance at 600 nm, bacteria were washed and resuspended in sterile PBS to a final concentration of 10^6^ CFU/ml. Fish were then intraperitoneally injected with 100 µl (10^5^ CFU) of bacterial suspension. Samples were collected after 1, 2, 3, 4, 7 and 10 days post-infection.

#### Infection and Peptide Administration

Fish were first infected with *P. damselae*, followed by administration of either hamp1 or hamp2 peptides 24 hours later, as previously described. Samples were collected after 1, 2, 3, 4, 7 and 10 days post-infection.

#### Peptide Administration and Infection

Fish were first injected with either hamp1 or hamp2 peptides, followed by infection with *P. damselae* 24 hours later, as previously described. Samples were collected after 1, 2, 3, 4, 7 and 10 days post-infection.

#### Controls

Fish were intraperitoneally injected with 100 µl of sterile PBS.

### Hematological Parameters and Tissue Iron Content

For determination of hematological parameters, 100 µl of blood were used in a 1:1 dilution with EDTA (1:10 diluted in sterile PBS) (BD Biosciences, San Jose CA, USA). For determination of serum parameters, non-heparinized blood was transferred into 1.5 ml microcentrifuge tubes, allowed to clot for 4 h at 4°C, and centrifuged at 16000 *×* g until a clear serum was obtained. Hematocrit was determined with microcappilaries, red blood cells were counted with an automated cell counter (Countess Automated Cell Counter, Invitrogen) and manually confirmed, and serum iron was blindly determined by a certified laboratory (CoreLab, Centro Hospitalar do Porto, Portugal). Non-heme iron was measured in livers by the bathophenanthroline method (37). Briefly, liver samples with an average weight of 100 mg were placed in iron-free Teflon vessels (ACV-Advanced Composite Vessel, CEM Corporation, Matthews NC, USA) and dried in a microwave oven (MDS 2000, CEM Corporation). Subsequently, dry tissue weights were determined and samples digested in an acid mixture (30% hydrochloric acid and 10% trichloroacetic acid) for 20 h at 65°C. After digestion, a chromogen reagent (5 volumes of deionized water, 5 volumes of saturated sodium acetate and 1 volume of 0.1% bathophenanthroline sulfonate/1% thioglycolic acid) was added to the samples in order to react with iron and obtain a colored product that was measured spectrophotometrically at 535 nm. The extinction coefficient for bathophenanthroline is 22.14 mM^-1^cm^-1^.

### RNA Isolation and cDNA Synthesis

Total RNA was isolated from tissues with the NZY Total RNA Isolation kit protocol for tissue samples (NZYtech, Lisboa, Portugal) with the optional on-column DNase treatment, according to the manufacturer’s instructions. Total RNA quantification was performed using a NanoDrop 1000 spectrophotometer (Thermo Fisher Scientific), and quality was assessed by running the samples in an Experion Automated Electrophoresis Station (Bio-Rad, Hercules, CA). For all samples, 2.5 µg of each were converted to cDNA using the NZY First-Strand cDNA Synthesis Kit (NZYTech) according to the manufacturer’s protocol.

### Gene Expression Analysis

Relative levels of *hamp1*, *hamp2*, *fpn1*, *fth* and s*lc11a2alpha* RNA were quantified by real-time PCR analysis using a CFX384 Touch Real-Time PCR Detection System (Bio-Rad). A total of 1 µL of each cDNA sample was added to a reaction mix containing 7.5 µL iTaq Universal SYBR Green Supermix (Bio-Rad), 5 µL double distilled H_2_O, and 250 nM of each primer (**Table 1**), making a total volume of 15 µL per reaction. A non-template control was included for each set of primers. The cycling profile was the following: 95°C for 3.5 min, 40 cycles of 95°C for 20 s and 59°C for 20 s. Samples were prepared in duplicates, a melting curve was generated for every PCR product to confirm the specificity of the assays, and a dilution series was prepared to check the efficiency of the reactions. Beta-actin (*actb*) was used as the housekeeping gene (M-value 0.177) (selected as the most stable gene among a suite of 5 candidates using the Delta CT method, Normfinder and Genorm, through RefFinder, http://blooge.cn/RefFinder/) (38). The comparative CT method (2^-ΔΔCT^ method) based on cycle threshold values was used to analyze gene expression levels.

**Table 1:** Primers used for gene expression analysis.

		FOR (5’ → 3’)	REV (5’ → 3’)
*Actin, beta*	*actb*	CAGAAGGACAGCTACGT	GTCATCTTCTCCCTGTTGGC
*Hepcidin 1*	*hamp1*	CATTGCAGTTGCAGTGACACT	CAGCCCTTGTTGCCTCTG
*Hepcidin 2*	*hamp2*	CTGCTGTCCCAGTCACTGA	ACCACATCCGCTCATATTAGG
*Ferroportin*	*fpn1*	GGCCTACTACAACCAGAACAT	AGGCCGCACTTCTTGCGAA
*Ferritin H*	*fth*	AACCATGAGTTCTCAGGTGAG	TTAGCTGCTCTCTTTGCCCAG
*Solute Carrier Family 11 Member 2, alpha*	*slc11a2alpha*	CGCGTTCAACCTCCTCTCCTCT	AGCCCTCGCAGTACGGCACA

### Statistical Analysis

Statistical analysis was carried out using GraphPad Prism 8 (GraphPad Software Inc, San Jose CA, USA). Multiple comparisons were performed with One-way ANOVA and *post hoc* Student Newman-Keuls test. A *p* value <0.05 was considered statistically significant.

## Results

### Hamp1, but Not Hamp2, Has a Significant Impact on the Iron Status of Sea Bass

Before we could evaluate the prophylactic or therapeutic potential of either hamp1 or hamp2 to treat infectious diseases and iron disorders, we would need to understand their impact on the iron status of healthy animals. To assess that effect, we administered either hamp1 or hamp2 alone, and then evaluated blood parameters that are influenced by iron availability, such as hematocrit and red blood cells number, iron mobilization by looking into serum iron levels, and also the expression of various iron related genes, such as hepcidin itself, its target, the sole known iron exporter ferroportin, as well as the iron storage protein ferritin and the solute carrier family 11 member 2 alpha.

**Figure 2:**
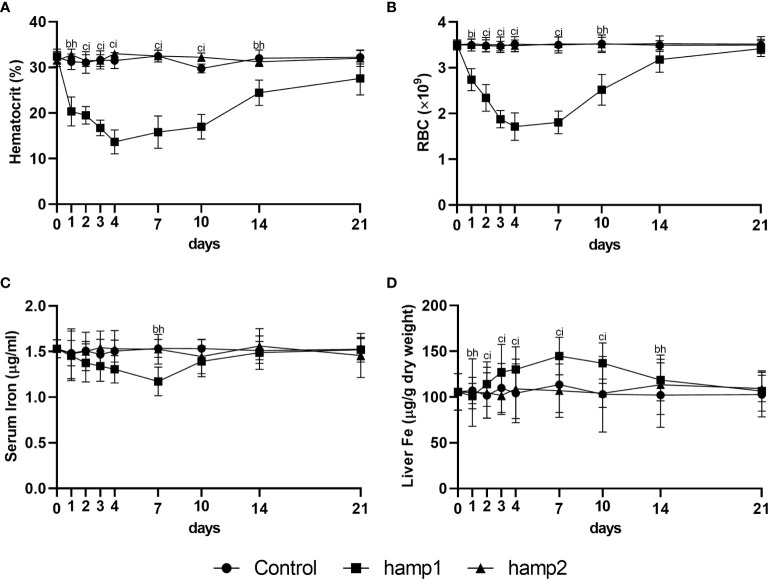
Hematological and serological parameters and tissue iron content in sea bass administered hamp1 or hamp2 peptides. **(A)** hematocrit; **(B)** red blood cell (RBC) number; **(C)** serum iron; **(D)** liver iron. Values are expressed as means ± standard deviation (n=5). Differences among groups were considered significant at *p<*0.05, *p<*0.01, and *p<*0.001, represented respectively by the letters a, b, c between control and hamp1, d, e, f between control and hamp2 and g, h, i between hamp1 and hamp2.

Administration of hamp1 to healthy sea bass led to a drastic decrease in both the hematocrit (**Figure 2A**) and number of red blood cells (RBC) (**Figure 2B**), effectively leading to a condition of anemia. These decreases occurred very steeply up to day 4 post-administration, after which a gradual recovery could be seen, returning to near normal levels after 21 days. This was also accompanied by a gradual decrease in serum iron levels (**Figure 2C**), and an increase in iron accumulation in the liver (**Figure 2D**), with both parameters peaking at day 7 followed by steady returns to normal levels. Administration of hamp2 had no significant effects on any of these parameters (**Figures 2A–D**).

**Figure 3:**
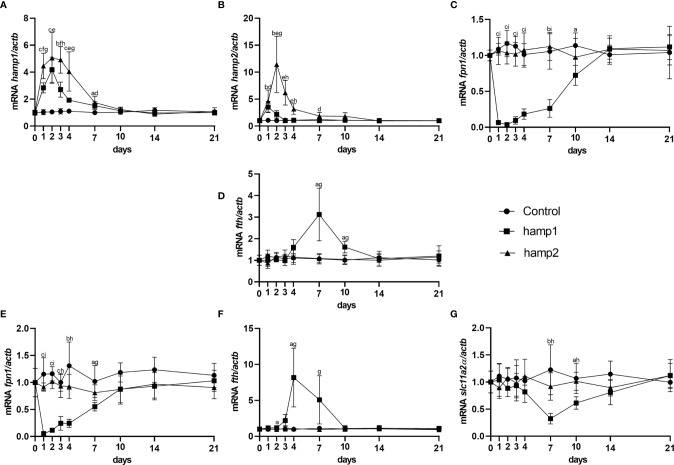
Gene expression in the liver and intestine at 1, 2, 3, 4, 7, 10, 14 and 21 days after hamp1 or hamp2 peptide administration. **(A)** *hamp1*, **(B)** *hamp2*, **(C)** *fpn1*, **(D)** *fth* expression in the liver; **(E)** *fpn1*, **(F)** *fth*, ***(*G*)*** *slc11a2alpha* expression in the intestine of peptide administered (hamp1/2) sea bass. *Actb* was used as the housekeeping gene. Values are expressed as means ± standard deviation (n=5). Differences among groups were considered significant at *p<*0.05, *p<*0.01, and *p<*0.001, represented respectively by the letters a, b, c between control and hamp1, d, e, f between control and hamp2 and g, h, i between hamp1 and hamp2.

When looking at gene expression in the liver, administration of hamp1 led to early increases in both *hamp1* and *hamp2* expression (**Figures 3A, B**). It also led to a very significant reduction in *fpn* expression (**Figure 3C**), as early as day 1 post-administration, followed by a slow but gradual recovery up to 21 days. Ferritin expression levels were found to be increased at day 7 (coinciding with the peak of iron accumulation in the liver), followed by a recovery towards day 21, but still above normal levels at day 10 (**Figure 3D**). In the intestine, *fpn1* was similarly downregulated (**Figure 3E**), although with a slightly faster recovery to normal levels than in the liver (day 10 *vs*. day 14). This was accompanied by significant increases in *fth* expression at days 4 and 7 (**Figure 3F**) and decreases in the expression of slc11a2alpha at days 7 and 10 (**Figure 3G**). Administration of hamp2 also led to significant early increases in the expression of both *hamp1* and *hamp2* in the liver (**Figures 3A, B**) but had no impact in the expression of any other tested genes, either in the liver or intestine (**Figures 3C–G**).

### Hamp1 Both Attenuates and Potentiates the Various Effects of Iron Overload

To test the potential of hepcidin to treat iron disorders, we performed a simple model of iron overload (to serve as a baseline for comparison), as well as a model of iron overload followed by administration of either hamp1 or hamp2 (despite earlier results suggesting a limited involvement of hamp2 in iron metabolism). Furthermore, since hamp1 has a significant impact on the iron status of healthy animals, leading to anemia, a model of peptide administration followed by iron overload was tested but not pursued, as the animals would be in a debilitated state and subsequent iron overload was found to cause further damaging effects.

**Figure 4:**
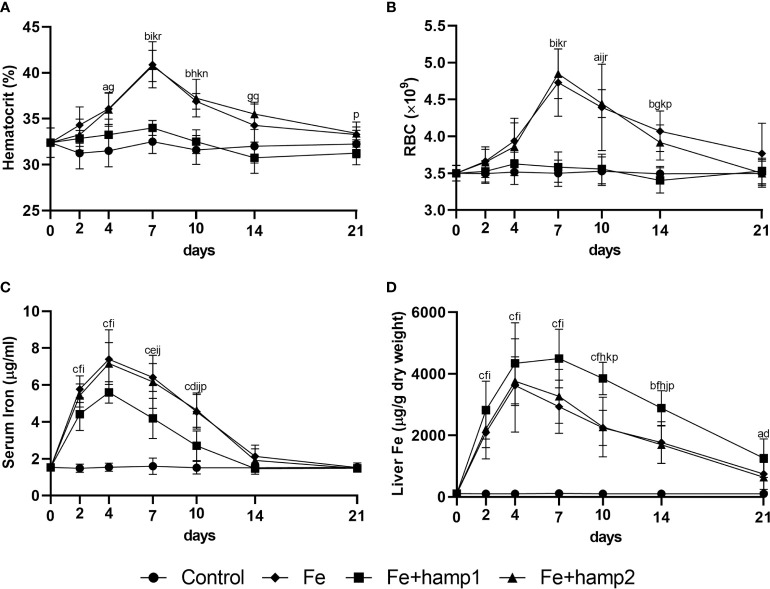
Hematological and serological parameters and tissue iron content in sea bass after experimental iron overload (Fe) or iron overload and peptide administration (Fe+hamp1/2). **(A)** hematocrit; **(B)** red blood cell (RBC) number; **(C)** serum iron; **(D)** liver iron. Values are expressed as means ± standard deviation (n=5). Differences among groups were considered significant at *p<*0.05, *p<*0.01, and *p<*0.001, represented respectively by the letters a, b, c between control and Fe animals, d, e, f between control and Fe+hamp1, g, h, i between control and Fe+hamp2, j, k, l between Fe and Fe+hamp1, m, n, o between Fe and Fe+hamp2 and p, q, r between Fe+hamp1 and Fe+hamp2.

Iron overload alone led to increases in hematocrit, RBC numbers, liver iron accumulation and serum iron levels (**Figure 4**), with all parameters peaking mostly at 7 days post-overload (barring serum iron, that peaked at 4 days), followed by gradual decreases towards normal levels up to day 21, but with some parameters still slightly elevated at that time point (namely, hematocrit and liver iron). Iron overload followed by hamp2 administration had comparable effects to iron overload alone in all of these parameters, not differing significantly in any of them.

However, when hamp1 is administered after iron overload, no significant increases are observed in either hematocrit or RBC numbers. However, there are still increases in serum iron, although slightly lower than in the other experimental groups, and in liver iron content, in this case significantly higher than in the other experimental groups (**Figure 4**).

**Figure 5:**
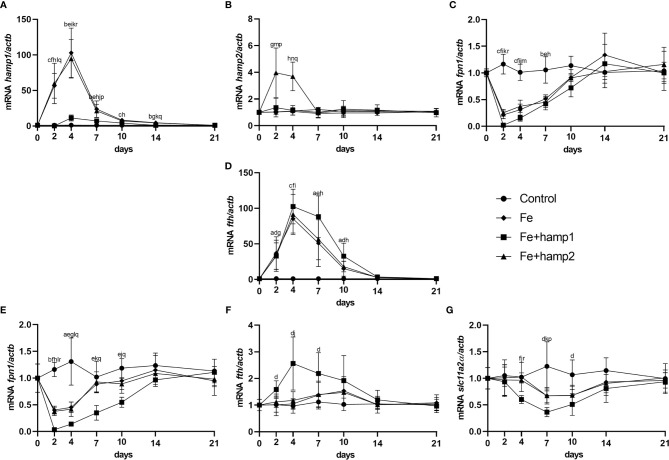
Gene expression in the liver and intestine at 2, 4, 7, 10, 14 and 21 days after experimental iron overload and peptide administration. **(A)** *hamp1*, **(B)** *hamp2*, **(C)** *fpn1*, **(D)** *fth* expression in the liver; **(E)** *fpn1*, **(F)** *fth*, **(G)** *slc11a2alpha* expression in the intestine of iron overload (Fe) and iron overload and peptide administered (Fe+hamp1/2) sea bass. *Actb* was used as the housekeeping gene. Values are expressed as means ± standard deviation (n=5). Differences among groups were considered significant at *p<*0.05, *p<*0.01, and *p<*0.001, represented respectively by the letters a, b, c between control and Fe animals, d, e, f between control and Fe+hamp1, g, h, i between control and Fe+hamp2, j, k, l between Fe and Fe+hamp1, m, n, o between Fe and Fe+hamp2 and p, q, r between Fe+hamp1 and Fe+hamp2.

Both iron overload alone and iron overload followed by hamp2 administration mostly caused similar effects on gene expression. We observed a high increase in *hamp1* in both experimental groups (**Figure 5A**), but only administration of hamp2 caused a further increase in *hamp2* expression in the liver (**Figure 5B**). Similar increased patterns of expression were observed for *fth* in the liver (**Figure 5D**), with no changes in the intestine (**Figure 5F**), as well as of decreased expression of *fpn1* both in the liver and intestine (**Figures 5C, E**), and *slc11a2alpha* in the intestine (**Figure 5G**). Once again, iron overload followed by administration of hamp1 had different effects, causing either dissimilar or more pronounced changes in gene expression. Only a minor increase in *hamp1* expression was observed, with no significant changes in *hamp2* (**Figures 5A, B**). Stronger decreases in *fpn1* (**Figures 5C, E**) and *slc11a2alpha* (**Figure 5G**) expressions were also observed, as well as higher increases in *fth* expression (**Figures 5D, F**), both in the liver and intestine, possibly indicating a much more pronounced limitation in iron release and absorption.

### Hamp2 Has a Significant Protective Affect Against Infection With *P. damselae*

In order to investigate the potential of hepcidin to prevent or treat bacterial diseases, we made experimental infections with the Gram-negative bacteria *Photobacterium damselae* spp. *piscicida*. Two major models were performed, one of infection followed by peptide administration (infected+hamp1/2 - therapeutic potential) and the other of peptide administration followed by infection (hamp1/2+infected - prophylactic potential), as well as a simple model of infection without any kind of peptide administration (as a baseline for comparison). Although with some differences, both models demonstrated that hamp2 is highly effective against *P. damselae*, whereas hamp1 is not only ineffective, but even slightly more deleterious.

**Figure 6:**
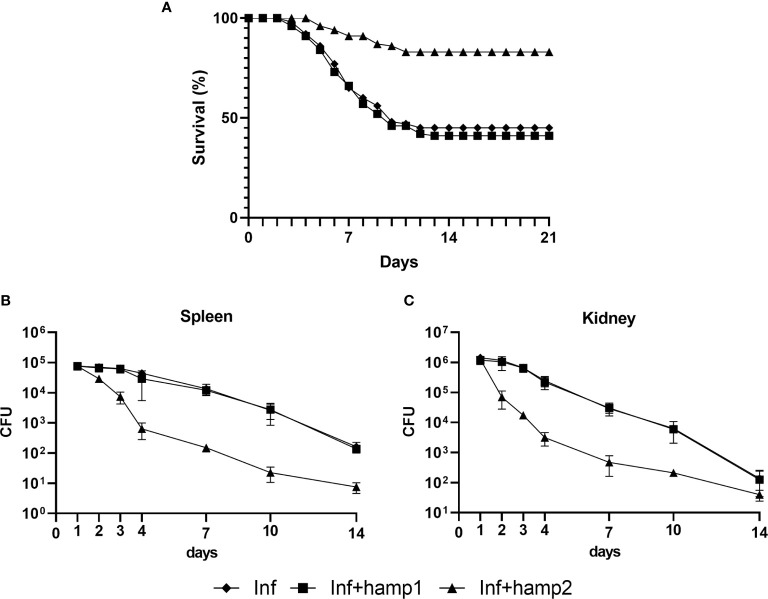
Survival curves and CFU counts in sea bass administered hepcidin 24 hours after experimental infection (post-infection). Mortality was assessed during 21 days of infection with *P. damselae*, followed by administration of either hamp1 or hamp2. Colony forming unit (CFU) values are expressed as means ± standard deviation (n=5).

In the model of infection followed by peptide administration, we could see a clear reduction in fish mortality derived from hamp2 administration, reduced from around 55% in the control infection to less than 17% in hamp2 administered animals (**Figure 6A**). Hamp1 on the other hand, not only it did not reduce mortality but in fact slightly increased it, to around 59%. Similar results can be seen for the CFU counts in both the spleen and head kidney, with much reduced bacterial loads in hamp2 administered animals, whereas no differences were observed between infected and infected+hamp1 animals (**Figures 6B, C**).

**Figure 7:**
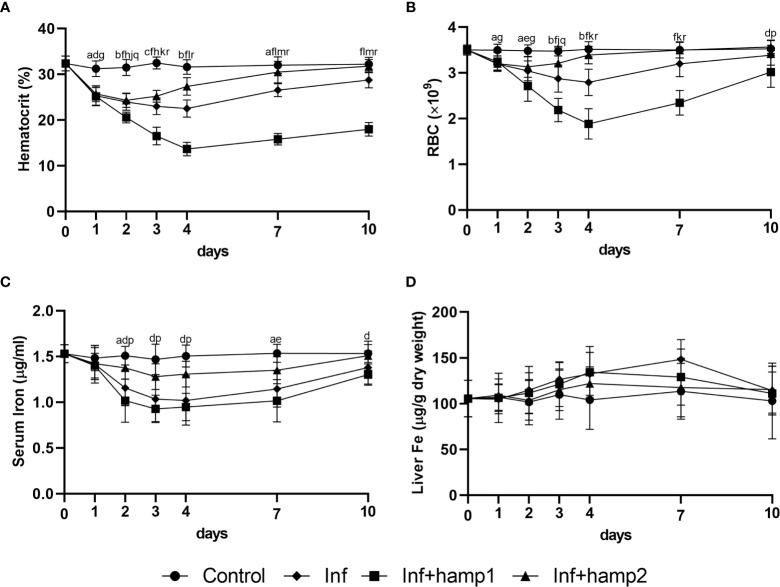
Hematological and serological parameters and tissue iron content in sea bass administered hepcidin 24 hours after experimental infection (post-infection). **(A)** hematocrit; **(B)** red blood cell (RBC) number; **(C)** serum iron; **(D)** liver iron. Values are expressed as means ± standard deviation (n=5). Differences among groups were considered significant at *p<*0.05, *p<*0.01, and *p<*0.001, represented respectively by the letters a, b, c between control and infected (Inf) animals, d, e, f between control and infected+hamp1 (Inf+hamp1), g, h, i between control and infected+hamp2 (Inf+hamp2), j, k, l between Inf and Inf+hamp1, m, n, o between Inf and Inf+hamp2 and p, q, r between Inf+hamp1 and Inf+hamp2.

Infection alone led to significant decreases in all hematological and serological parameters, up to 4 days post-infection, with gradual recovery to normal levels from day 7 forward (**Figures 7A–C**). These decreases were less pronounced with hamp2 administration, with a faster recovery to normal levels, but more pronounced with hamp1 administration, with higher decreases of the hematocrit, RBC numbers and serum iron levels, when compared with control infection. No significant changes were observed for liver iron content in any of the experimental groups, although a slight tendency for increase could be seen in solely infected animals (**Figure 7D**).

**Figure 8:**
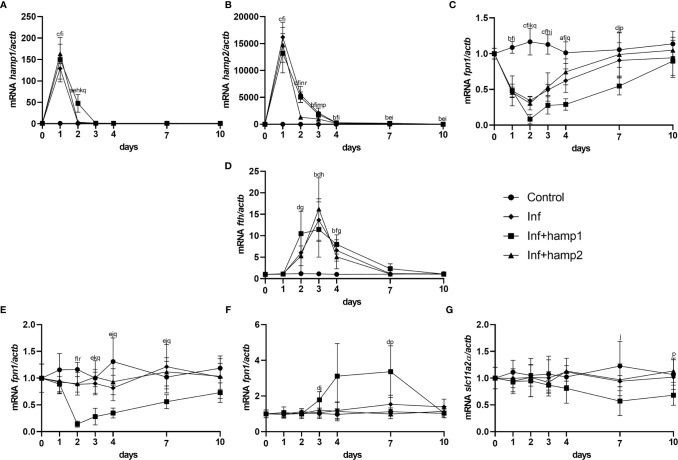
Gene expression in the liver and intestine at 1, 2, 3, 4, 7 and 10 days, in sea bass administered hepcidin 24 hours after experimental infection (post-infection). **(A)** *hamp1*, **(B)** *hamp2*, **(C)** *fpn1*, **(D)** *fth* expression in the liver; **(E)** *fpn1*, **(F)** *fth*, **(G)** *slc11a2alpha* expression in the intestine of infected (Inf) and infected and peptide administered (Inf+hamp1/2) sea bass. *Actb* was used as the housekeeping gene. Values are expressed as means ± standard deviation (n=5). Differences among groups were considered significant at *p<*0.05, *p<*0.01, and *p<*0.001, represented respectively by the letters a, b, c between control and Inf animals, d, e, f between control and Inf+hamp1, g, h, i between control and Inf+hamp2, j, k, l between Inf and Inf+hamp1, m, n, o between Inf and Inf+hamp2 and p, q, r between Inf+hamp1 and Inf+hamp2.

Looking at gene expression, in the liver of infected animals a significant increase in *hamp1* expression is observed at day 1, quickly reverting to near normal levels at day 2 (**Figure 8A**). *Hamp2* also sees the highest increase at day 1, and gradually decreases up to day 10, but still kept slightly overexpressed (**Figure 8B**). Increases in *fth* can also be, peaking at 3 days post-infection, whereas *fpn1* gradually decreases up to 2 days, then slowly returns to normal levels (**Figures 8C, D**). In the intestine, no significant changes could be seen in the expression of any of the tested genes (**Figures 8E–G**). In infected+hamp1 animals, similar patterns of expression can be observed in the liver (**Figures 8A–D**), with similar increases in *hamp1*, *hamp2* and *fth* expression, although *hamp1* levels are kept elevated up to day 2, rather than just day 1. *Fpn1* suffers an even more pronounced downregulation, reaching lower levels and recovering slower. However, in the intestine, significant changes can be observed for all genes (**Figures 8E–G**), with a downregulation of both *fpn1* and *slc11a2alpha*, in the earlier and late days of infection, respectively, and an up regulation of *fth*. Lastly, in infected+hamp2 animals, patterns of gene expression in both the liver and intestine were similar to the ones observed for infected animals, with one notable exception: *hamp2* levels in the liver still peaked at day 1 post-infection but decreased more rapidly at day 2 (around 5 times lower than infected and infected+hamp1) (**Figures 8B**).

**Figure 9:**
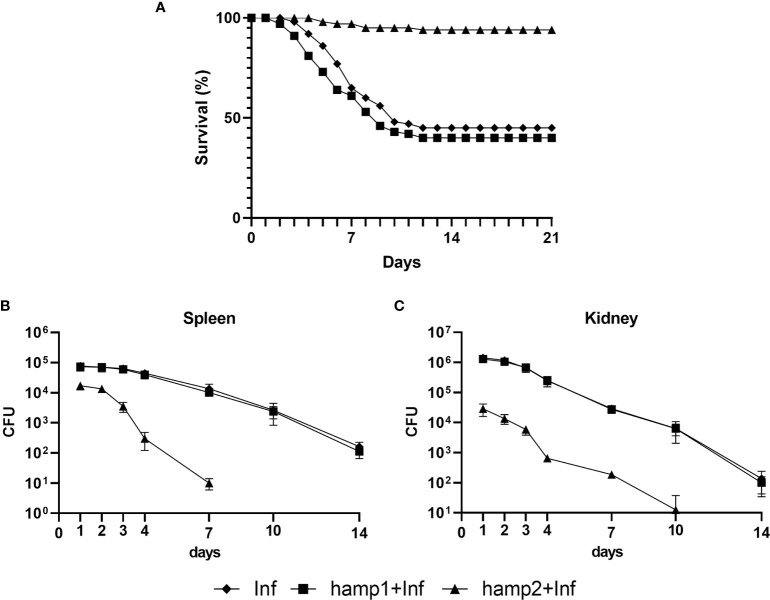
Survival curves and CFU counts of sea bass administered hepcidin 24 hours before experimental infection (pre-infection). Mortality was assessed during 21 days of infection with *P. damselae*, preceded by administration of either hamp1 or hamp2. Colony forming unit (CFU) values are expressed as means ± standard deviation (n=5).

In our final experimental model, of peptide administration followed by infection, mortality was greatly reduced by the pre-administration of hamp2, from the 55% of the control infection to a mere 6% (**Figure 9A**). Once again, hamp1 not only did not reduce mortality but increased it slightly more, to around 60%. Results for the CFU counts were even more expressive, with rapidly decreasing bacterial loads in hamp2 administered animals, to the point where no CFUs could be counted after 7 and 10 days of infection in the spleen and head kidney, respectively (**Figures 9B, C**). Again, no significant differences were observed between infected and hamp1+infected animals.

**Figure 10:**
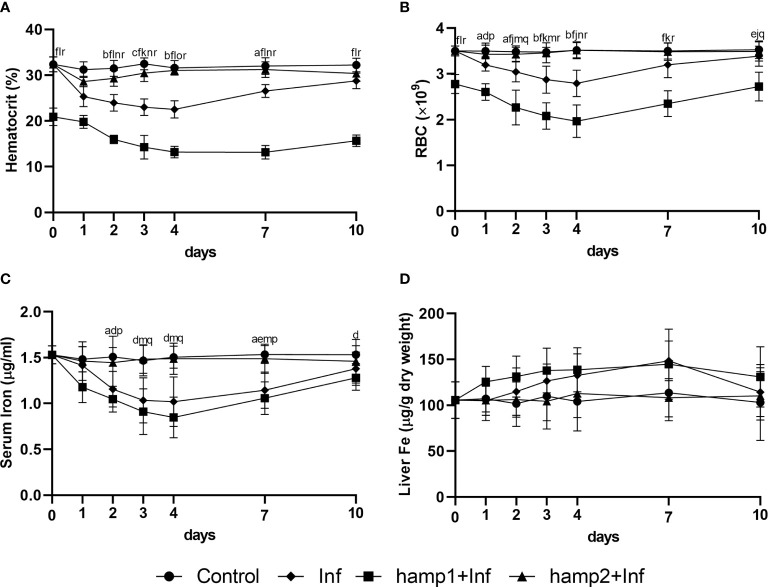
Hematological, serological and tissue iron content in sea bass administered hepcidin 24 hours before experimental infection (pre-infection). **(A)** hematocrit; **(B)** red blood cell (RBC) number; **(C)** serum iron; **(D)** liver iron. Values are expressed as means ± standard deviation (n=5). Differences among groups were considered significant at *p<*0.05, *p<*0.01, and *p<*0.001, represented respectively by the letters a, b, c between control and Inf animals, d, e, f between control and hamp1+Inf, g, h, i between control and hamp2+Inf, j, k, l between Inf and hamp1+Inf, m, n, o between Inf and hamp2+Inf and p, q, r between hamp1+Inf and hamp2+Inf.

Variations in hematological and serological parameters were mostly comparable to the previous experimental model of infection, but with a clear difference at day 0, where hematocrit and RBC numbers were already significantly reduced in hamp1 administered animals. This is not unexpected since in this experiment this day would be equivalent to day 1 post-peptide administration (**Figure 2**). Decreases were observed up to 4 days post-infection and gradual recovery to normal levels from day 7 forward (**Figures 10A–C**). Again, these decreases were more pronounced with hamp1 administration and less pronounced with hamp2 administration, when compared with control infection, and no significant changes were observed for liver iron content (**Figure 10D**).

**Figure 11:**
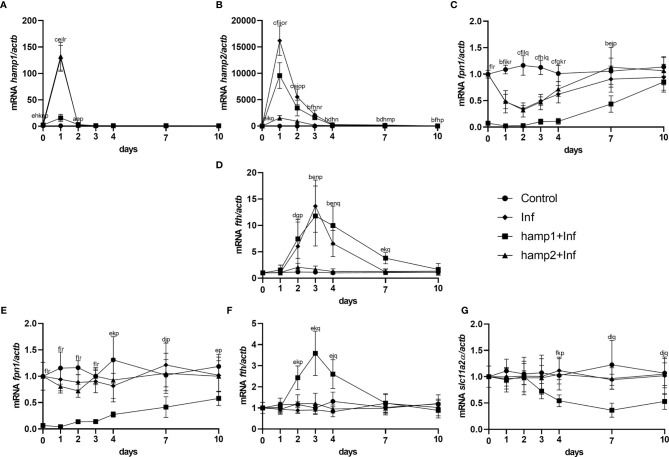
Gene expression in the liver and intestine at 1, 2, 3, 4, 7 and 10 days, in sea bass administered hepcidin 24 hours before experimental infection (pre-infection). **(A)** *hamp1*, **(B)** *hamp2*, **(C)** *fpn1*, **(D)** *fth* expression in the liver; **(E)** *slc11a2alpha*, **(F)** *fpn1*, **(G)** *fth* expression in the intestine of infected (Inf) and peptide administered and infected (hamp1/2+Inf) sea bass. *Actb* was used as the housekeeping gene. Values are expressed as means ± standard deviation (n=5). Differences among groups were considered significant at *p<*0.05, *p<*0.01, and *p<*0.001, represented respectively by the letters a, b, c between control and Inf animals, d, e, f between control and hamp1+Inf, g, h, i between control and hamp2+Inf, j, k, l between Inf and hamp1+Inf, m, n, o between Inf and hamp2+Inf and p, q, r between hamp1+Inf and hamp2+Inf.

Gene expression patterns were also very similar to the ones observed for the previous infection model, but nevertheless with several significant differences. *Hamp1* expression was found to be increased in response to both hamp1 and hamp2 administration, but with a much more limited increase in response to hamp1 (**Figure 11A**). Similarly, *hamp2* also responded to the administration of either peptide, but to a much lesser extent to hamp2 (**Figure 11B**). Also, at day 0, both hepcidin types are already slightly overexpressed in the peptide administered groups, when compared with infection alone, again reminiscent of day 1 of the peptide administration experiment (**Figure 3**). Similarly, *fpn1* levels were already much lower than normal at day 0, both in the liver and intestine, but followed a similar pattern of decline and subsequent recovery (**Figures 11C, E**). *Fth* levels were found to be elevated in both the liver and intestine (**Figures 11D, F**), whereas *slc11a2alpha* levels in the intestine started decreasing significantly at day 4 post-infection, followed by a recovery towards normal levels, but still under expressed after 10 days (**Figure 11G**).

## Discussion

Diseases are a major problem in aquaculture, every year causing significant production and economic losses. Some preventive measures can be taken, mostly reliant on vaccination strategies, but there is a lack of effective commercial vaccines for a large number of pathogens. Therapeutic measures can also be applied, but usually too late and are mostly based on antibiotics, the use of which is becoming increasingly problematic, due to increased pathogen multiresistance, toxicity, inability to treat viral diseases and the possibility of entering the human food chain (39, 40). As such, it has become crucial to find efficient therapeutic alternatives to replace or reduce the use of antibiotics. Antimicrobial peptides are a promising alternative to the use of antibiotics and other chemical compounds (7, 9, 41, 42) presenting several advantages, such as limited toxic effects and broad spectrum of antimicrobial properties against bacteria, as well as viruses, fungi, parasites and even anomalous cells. They are also less prone to cause bacterial resistance, but still not totally impervious to that effect (43–46). Among antimicrobial peptides, hepcidin is of particular interest, especially in teleost fish. In mammals, hepcidin is present as a single gene (with the mouse being the sole exception, so far) (12, 13, 19, 20), and although originally characterized as an antimicrobial peptide, that function was quickly relegated to the background. Currently, hepcidin is considered the *de facto* key regulator of iron metabolism, due to its interaction with the only known iron exporter, ferroportin (15, 47–49). This effectively limits the applications of mammalian hepcidin mostly to the treatment of iron disorders, by targeting the hepcidin/ferroportin axis, since its use in infections would have a severe impact in several iron related parameters, with negative consequences to the host. However, many teleost fish (23, 24, 28, 50, 51), sea bass included (26), present two different types of hepcidin, with different functions: type 1 hepcidin, very similar to mammalian hepcidin and also with a role in iron metabolism, and one to several type 2 hepcidins, with almost exclusively antimicrobial roles. As such, contrary to mammalian hepcidin, fish hepcidins have the potential to be differentially applied in the treatment or prevention of iron disorders and infections. Although some studies exist on the antimicrobial potential of fish derived hepcidins, information about their possible applications in iron disorders is lacking.

In order to determine the potential applications of sea bass derived hepcidins, in this study, we have performed several experimental models of both iron overload and infection, where we administered two different peptides, hamp1 and hamp2, and evaluated their effects on several parameters, including iron levels, hematological parameters and expression of iron metabolism-related relevant genes.

The impact of administration of hepcidin in healthy animals has shown that hamp1 had a significant impact on several hematological parameters, causing decreases in both hematocrit and red blood cell numbers, and thus leading to a condition of severe anemia. The role of type 1 hepcidins is well established, with its major target being the iron exporter ferroportin (27, 47). When hepcidin levels are increased, it binds to ferroportin, causing its internalization and degradation in various cell types, most relevant of all the hepatocytes, the major place for iron storage, the reticuloendothelial macrophages, deeply involved in hemocateresis and the intestinal enterocytes, responsible for iron absorption, effectively blocking any kind of iron release from these cells. A biological increase in hamp1 levels is known to occur in response to conditions of iron overload, to signal the body to limit further iron absorption (16, 17, 52), as well as during various infectious diseases, as a mechanism to limit pathogen proliferation (26, 53–55). An increase in the expression of *hamp1* has an inhibitory effect on ferroportin, limiting its expression and membrane presence, and thus limiting iron release from hepatocytes, macrophages and enterocytes. Furthermore, the accumulation of iron in the intestinal enterocytes, in the form of ferritin, will also lead to a suppression of slc11a2-mediated iron uptake, decreasing iron absorption (56–58). During infections, this mechanism is responsible for limiting iron availability for pathogens, effectively starving them of this essential nutrient. However, this is a double edged sword, since iron is not only unavailable for pathogens, but also for several of the host’s processes, including erythropoiesis. This means that, during prolonged infections, erythrocytes are still being recycled, but since there is no iron available, erythropoiesis comes to a standstill, thus leading to a gradual decrease in the number of red blood cells and hematocrit, causing a condition commonly referred to as anemia of inflammation or anemia of chronic disease. When administered to healthy animals, it simply leads to anemia and to a possible debilitative state (while not causing mortality, the animals were generally more lethargic and less prone to feed). On the other hand, administration of hamp2 to healthy animals had no visible effects on any of the evaluated parameters, once again opening the door to the differential application of these peptides.

Following these observations, we then introduced a new variable, iron overload. Iron overload alone led to alterations in the various measured hematological parameters, with significant increases in red blood cell numbers and hematocrit, as well as circulating iron levels and liver iron accumulation. It is well known that iron deficiency or anemia leads to a decrease in hematological parameters and as such, it is frequently assumed that iron overload would lead to a reverse situation, of increased hematological parameters. This is not so straightforward, as in many cases iron overload can also cause no changes or even lead to anemia [such as some forms of beta thalassemia or hypochromic microcytic anemia (59, 60)], but we have previously observed that in sea bass, during iron overload and despite the associated increase in *hamp1* expression, hematological parameters are often increased (58, 61), and that can also be seen here. It is likely an additional mechanism is triggered to cope with the excess of iron, by using more in the production of hemoglobin and red blood cells and thus, keeping it in a non-free, non-toxic form, but why and how exactly this occurs remains unknown. Similarly, gene expression profile is in accordance with an attempt to increase iron storage and limit iron release and absorption, denoted by the decreased *fpn1* and *slc11a2alpha* expression, and further increased *hamp1* expression, as well as of *fth*. Since we had already observed that hamp2 has no effect on iron metabolism (26), we expected a similar pattern of changes in animals administered both iron dextran and the hamp2 peptide, which was what we observed. The only significant difference when compared with iron overloaded animals was an increase in *hamp2* expression, likely induced by hamp2 itself, as neither iron nor hamp1 administration seem to produce a similar effect.

However, iron overload followed by administration of hamp1 had a significantly different outcome. There were no discernible changes in either hematological parameter, circulating serum iron levels were slightly less elevated and liver iron accumulation significantly higher throughout the duration of the experiment, which seems to indicate a higher rate of iron retention, limiting the increased erythropoiesis that occurs in iron overloaded animals. Gene expression also reflects this, with an even more aggressive suppression of *fpn1* and *slc11a2alpha* expression, as well as a higher *fth* expression. Contrary to hamp2, which itself induced an increase in its own expression, administration of hamp1 limited *hamp1* expression when compared to iron overload, or iron overload and hamp2 administered animals, indicating that the administered hamp1 levels were already high enough to deal with the increased iron levels. Taken together, all this data points towards a possible application of hamp1 in the treatment of iron overload, similar to mammalian hamp1 (62, 63). Sea bass hamp1 can even be used as a substitute to mammalian hepcidins since it is also known to be able to regulate mammalian ferroportin (27). The potential to prevent iron overload is less encouraging, with some preliminary tests showing that animals pre-treated with hamp1 before iron overload seemed to be become somewhat more susceptible, to point of having some mortality not seen in animals with only iron overload. It seems that any benefit derived from iron withholding is quickly undermined by the debilitative state derived from anemia, and probably leads to a less than ideal coping with iron toxicity, but this will require further studies.

Having explored the possible impact of hepcidin on iron metabolism, we then moved on to its other major function, the role during infection. Here, things are a little bit different between mammals and teleost fish. Although hepcidin was originally characterized in mammals as an antimicrobial peptide (12, 13), that function is now considered very minor, with hepcidin assuming the role of key regulator of iron metabolism (14, 16, 17). During infection, its major function is to lead to iron withholding, to limit pathogen proliferation, with seemingly minimal antimicrobial activity. However, in teleost fish presenting two hepcidin types, these functions seem to be separated. Fewer studies exist pertaining the role of fish hepcidins in iron metabolism, but there are some reports showing that type 1 hepcidins (characterized by the presence of the hypothetical iron regulatory sequence Q-S/I-H-L/I-S/A-L in the N-terminal region) seem to be mostly involved in iron regulation, likely limiting iron availability for pathogens (26, 27, 51, 64, 65). On the other hand, type 2 hepcidins (lacking the iron regulatory sequence) are highly diverse and are considered to have a mostly antimicrobial activity, responding to a wide variety of pathogens, including bacteria, fungi, viruses and parasites (26, 28–30, 33, 66–69), and even to anomalous cells (70, 71). In both cases we say mostly because, due the huge diversity in teleost fish hepcidins, there is the occasional report indicating some potential antimicrobial activity for hamp1 or a small role in iron regulation for hamp2, as well as reports where hepcidins were wrongly characterized as a different type, and as such, attributed an erroneous function. To complicate matters even more, in teleost fish with a single hepcidin (type 1, such as cyprinids and salmonids), the antimicrobial activity can be very significant, asides from its role in iron metabolism (35, 72–76). Nevertheless, there are various functional studies testing the effects of hepcidin peptides on a diversity of pathogens, mostly in *in vitro* conditions (28–32, 77–80), but a few also *in vivo* (28, 33, 34, 36), with most results pointing towards a much higher diversity (in amino acid composition and cysteine number) and antimicrobial activity of type 2 hepcidins. We have also previously characterized both hamp1 and various hamp2 in sea bass, and tested them in *in vitro* conditions, with hamp1 has showing little to no antimicrobial activity, whereas the various hamp2 have shown differential activity against Gram-negative and Gram-positive bacteria (26), and based on those results, we have selected the most promising hamp2, the one with highest antimicrobial activity, for further testing in the *in vivo* experimental infections of this work.

We took two different approaches to infection: a therapeutic study, where we administered hepcidin after infection, and a prophylactic study, where we administered hepcidin before infection, to evaluate both the potential to treat and prevent bacterial diseases, in this case pasteurelosis, caused by *Photobacterium damsela* spp. *piscicida*. The outcomes of both experiments were very similar, with clear conclusions. First, that hamp1 does not seem to help in the prevention or treatment of pasteurelosis and may actually make it worse as evidenced by the slight increase in animal mortality. Again, this is likely derived from the significant impact that hamp1 has on several hematological and iron parameters, leading to a condition of anemia that introduces a debilitative state and makes the animals more susceptible to infection. However, we should not completely exclude the usefulness of hamp1, even more so because we could observe a biological response of *hamp1* to infection (26). More in depth studies using lower doses of hamp1, which would have a more limited impact on iron metabolism, would have to be performed in order to evaluate its real potential, either alone or together with hamp2. The second conclusion is that hamp2 is highly effective against pasteurelosis, even more so when administered before infection, as indicated by the lower mortality and bacterial loads observed. These findings are also in agreement with a recent study from Álvarez et al. (36), that has shown that pre-administration of a type 2 hepcidin in sea bass can limit mortality caused by *Vibrio anguillarum*, reducing it from around 72% to less than 24%. Although seemingly not as effective in the prevention of vibriosis, when compared with pasteurelosis, those results are nevertheless very promising, if we take into consideration that *V. anguillarum* seems to be much more resistant to type 2 hepcidins than *P. damselae* (26). Additionally, as expected from previous results, hamp2 had no significant impact on iron metabolism and also hampered the development of anemia of inflammation, contributing for a better health status of the animals.

In summary, we have shown that the administration of the two sea bass hepcidins types elicit different responses, with hamp1 impacting in the regulation of iron metabolism and hamp2 having a very significant protective activity against bacterial infections. Hamp2 apart from having a direct antimicrobial activity, may also be involved in immunomodulatory processes (81–83) and the inflammatory response, but further studies will be required to address this matter. Nevertheless, the doors are clearly open for the potential application of sea bass derived hepcidins in the treatment of iron disorders and, more importantly, as viable substitutes for the use of antibiotics in the prevention and treatment of infections, if we can overcome some of the current limitations for a wide use of antimicrobial peptides, such as costs and more effective ways of administration.

## Data Availability Statement

The original contributions presented in the study are included in the article/supplementary material. Further inquiries can be directed to the corresponding authors.

## Ethics Statement

The animal study was reviewed and approved by ORBEA Instituto de Ciências Biomédicas Abel Salazar.

## Author Contributions

CB designed and conducted experiments, analyzed data, and wrote the paper. JN designed, conducted and supervised the experiments, analyzed data, and wrote the paper. PR designed and supervised the experiments. PC, MN, and JG conducted experiments. JN had primary responsibility for final content. All authors contributed to the article and approved the submitted version.

## Funding

This work was funded by the structured program of R&D&I ATLANTIDA - Platform for the monitoring of the North Atlantic Ocean and tools for the sustainable exploitation of the marine resources (NORTE-01-0145-FEDER-000040), supported by the North Portugal Regional Operational Programme (NORTE2020), through the European Regional Development Fund (ERDF). CB is supported by a Ph.D. fellowship (SFRH/BD/114899/2016) financed by FCT - *Fundação para a Ciência e a Tecnologia/ Ministério da Ciência, Tecnologia e Ensino Superior*.

## Conflict of Interest

The authors declare that the research was conducted in the absence of any commercial or financial relationships that could be construed as a potential conflict of interest.

## Publisher’s Note

All claims expressed in this article are solely those of the authors and do not necessarily represent those of their affiliated organizations, or those of the publisher, the editors and the reviewers. Any product that may be evaluated in this article, or claim that may be made by its manufacturer, is not guaranteed or endorsed by the publisher.

## References

[1] Fao. The State of World Fisheries and Aquaculture 2020. Sustainability in Action. Rome: Food and Agriculture Organization of the United Nations (2020).

[2] Muniesa A Basurco B Aguilera C Furones D Reverté C Sanjuan-Vilaplana A. Mapping the Knowledge of the Main Diseases Affecting Sea Bass and Sea Bream in Mediterranean. Transboundary Emerging Dis (2020) 67:1089–100. doi: 10.1111/tbed.13482 31960605

[3] Lafferty KD Harvell CD Conrad JM Friedman CS Kent ML Kuris AM. Infectious Diseases Affect Marine Fisheries and Aquaculture Economics. Annu Rev Marine Sci (2015) 7:471–96. doi: 10.1146/annurev-marine-010814-015646 25251276

[4] Cabello FC Godfrey HP Tomova A Ivanova L Dölz H Millanao A. Antimicrobial Use in Aquaculture Re-Examined: Its Relevance to Antimicrobial Resistance and to Animal and Human Health. Environ Microbiol (2013) 15:1917–42. doi: 10.1111/1462-2920.12134 23711078

[5] Adams A. Progress, Challenges and Opportunities in Fish Vaccine Development. Fish Shellfish Immunol (2019) 90:210–4. doi: 10.1016/j.fsi.2019.04.066 31039441

[6] Browne K Chakraborty S Chen R Willcox MD Black DS Walsh WR. A New Era of Antibiotics: The Clinical Potential of Antimicrobial Peptides. Int J Mol Sci (2020) 21(19):7047. doi: 10.3390/ijms21197047 PMC7582481 32987946

[7] Chaturvedi P RaH B Pande A. Antimicrobial Peptides of Fish: Innocuous Alternatives to Antibiotics. Rev Aquacult (2020) 12:85–106. doi: 10.1111/raq.12306

[8] Valero Y Saraiva-Fraga M Costas B Guardiola FA. Antimicrobial Peptides From Fish: Beyond the Fight Against Pathogens. Rev Aquacult (2020) 12:224–53. doi: 10.1111/raq.12314

[9] Lei J Sun L Huang S Zhu C Li P He J. The Antimicrobial Peptides and Their Potential Clinical Applications. Am J Transl Res (2019) 11:3919–31. PMC6684887 31396309

[10] Masso-Silva JA Diamond G. Antimicrobial Peptides From Fish. Pharmaceuticals (2014) 7:265–310. doi: 10.3390/ph7030265 24594555 PMC3978493

[11] Katzenback BA. Antimicrobial Peptides as Mediators of Innate Immunity in Teleosts. Biol (Basel) (2015) 4:607–39. doi: 10.3390/biology4040607 PMC4690011 26426065

[12] Krause A Neitz S Magert HJ Schulz A Forssmann WG Schulz-Knappe P. LEAP-1, A Novel Highly Disulfide-Bonded Human Peptide, Exhibits Antimicrobial Activity. FEBS Lett (2000) 480:147–50. doi: 10.1016/S0014-5793(00)01920-7 11034317

[13] Park CH Valore EV Waring AJ Ganz T. Hepcidin, A Urinary Antimicrobial Peptide Synthesized in the Liver. J Biol Chem (2001) 276:7806–10. doi: 10.1074/jbc.M008922200 11113131

[14] Nicolas G Viatte L Bennoun M Beaumont C Kahn A Vaulont S. Hepcidin, A New Iron Regulatory Peptide. Blood Cells Mol Dis (2002) 29:327–35. doi: 10.1006/bcmd.2002.0573 12547223

[15] Nemeth E Tuttle MS Powelson J Vaughn MB Donovan A Ward DM. Hepcidin Regulates Cellular Iron Efflux by Binding to Ferroportin and Inducing Its Internalization. Science (2004) 306:2090–3. doi: 10.1126/science.1104742 15514116

[16] Nemeth E Ganz T. Regulation of Iron Metabolism by Hepcidin. Annu Rev Nutr (2006) 26:323–42. doi: 10.1146/annurev.nutr.26.061505.111303 16848710

[17] Viatte L Vaulont S. Hepcidin, the Iron Watcher. Biochimie (2009) 91:1223–8. doi: 10.1016/j.biochi.2009.06.012 19555735

[18] Ganz T. Hepcidin and Iron Regulation, 10 Years Later. Blood (2011) 117:4425–33. doi: 10.1182/blood-2011-01-258467 PMC3099567 21346250

[19] Pigeon C Ilyin G Courselaud B Leroyer P Turlin B Brissot P. A New Mouse Liver-Specific Gene, Encoding a Protein Homologous to Human Antimicrobial Peptide Hepcidin, Is Overexpressed During Iron Overload. J Biol Chem (2001) 276:7811–9. doi: 10.1074/jbc.M008923200 11113132

[20] Ilyin G Courselaud B Troadec MB Pigeon C Alizadeh M Leroyer P. Comparative Analysis of Mouse Hepcidin 1 and 2 Genes: Evidence for Different Patterns of Expression and Co-Inducibility During Iron Overload. FEBS Lett (2003) 542:22–6. doi: 10.1016/S0014-5793(03)00329-6 12729891

[21] Padhi A Verghese B. Evidence for Positive Darwinian Selection on the Hepcidin Gene of Perciform and Pleuronectiform Fishes. Mol Divers (2007) 11:119–30. doi: 10.1007/s11030-007-9066-4 18060573

[22] Hilton KB Lambert LA. Molecular Evolution and Characterization of Hepcidin Gene Products in Vertebrates. Gene (2008) 415:40–8. doi: 10.1016/j.gene.2008.02.016 18395368

[23] Cho YS Lee SY Kim KH Kim SK Kim DS Nam YK. Gene Structure and Differential Modulation of Multiple Rockbream (*Oplegnathus Fasciatus*) Hepcidin Isoforms Resulting From Different Biological Stimulations. Dev Comp Immunol (2009) 33:46–58. doi: 10.1016/j.dci.2008.07.009 18761369

[24] Martin-Antonio B Jimenez-Cantizano RM Salas-Leiton E Infante C Manchado M. Genomic Characterization and Gene Expression Analysis of Four Hepcidin Genes in the Redbanded Seabream (*Pagrus Auriga*). Fish Shellfish Immunol (2009) 26:483–91. doi: 10.1016/j.fsi.2009.01.012 19340950

[25] Mu Y Huo J Guan Y Fan D Xiao X Wei J. An Improved Genome Assembly for Larimichthys Crocea Reveals Hepcidin Gene Expansion With Diversified Regulation and Function. Commun Biol (2018) 1:195. doi: 10.1038/s42003-018-0207-3 30480097 PMC6240063

[26] Neves JV Caldas C Vieira I Ramos MF Rodrigues PN. Multiple Hepcidins in a Teleost Fish, *Dicentrarchus Labrax*: Different Hepcidins for Different Roles. J Immunol (2015) 195:2696–709. doi: 10.4049/jimmunol.1501153 26268656

[27] Neves JV Ramos MF Moreira AC Silva T Gomes MS Rodrigues PNS. Hamp1 But Not Hamp2 Regulates Ferroportin in Fish With Two Functionally Distinct Hepcidin Types. Sci Rep (2017) 7:14793. doi: 10.1038/s41598-017-14933-5 29093559 PMC5665920

[28] Zhang J Yu LP Li MF Sun L. Turbot (*Scophthalmus Maximus*) Hepcidin-1 and Hepcidin-2 Possess Antimicrobial Activity and Promote Resistance Against Bacterial and Viral Infection. Fish Shellfish Immunol (2014) 38:127–34. doi: 10.1016/j.fsi.2014.03.011 24647314

[29] Xie J Obiefuna V Hodgkinson JW Mcallister M Belosevic M. Teleost Antimicrobial Peptide Hepcidin Contributes to Host Defense of Goldfish (*Carassius Auratus L.*) Against *Trypanosoma Carassii* . Dev Comp Immunol (2019) 94:11–5. doi: 10.1016/j.dci.2019.01.007 30659854

[30] Zheng L Li Y Wang J Pan Y Chen J Zheng W. Antibacterial and Antiparasitic Activities Analysis of a Hepcidin-Like Antimicrobial Peptide From *Larimichthys Crocea* . Acta Oceanol Sin (2020) 39:129–39. doi: 10.1007/s13131-020-1580-6

[31] Wang Y Liu X Ma L Yu Y Yu H Mohammed S. Identification and Characterization of a Hepcidin From Half-Smooth Tongue Sole *Cynoglossus Semilaevis* . Fish Shellfish Immunol (2012) 33:213–9. doi: 10.1016/j.fsi.2012.04.011 22565018

[32] Zhou JG Wei JG Xu D Cui HC Yan Y Ou-Yang ZL. Molecular Cloning and Characterization of Two Novel Hepcidins From Orange-Spotted Grouper, *Epinephelus Coioides* . Fish Shellfish Immunol (2011) 30:559–68. doi: 10.1016/j.fsi.2010.11.021 21145974

[33] Wang YD Kung CW Chen JY. Antiviral Activity by Fish Antimicrobial Peptides of Epinecidin-1 and Hepcidin 1-5 Against Nervous Necrosis Virus in Medaka. Peptides (2010) 31:1026–33. doi: 10.1016/j.peptides.2010.02.025 20214942

[34] Chen J Nie L Chen J. Mudskipper (*Boleophthalmus Pectinirostris*) Hepcidin-1 and Hepcidin-2 Present Different Gene Expression Profile and Antibacterial Activity and Possess Distinct Protective Effect Against *Edwardsiella Tarda* Infection. Probiotics Antimicrob Proteins (2018) 10:176–85. doi: 10.1007/s12602-017-9352-0 29151250

[35] Hu Y Kurobe T Liu X Zhang YA Su J Yuan G. Hamp Type-1 Promotes Antimicrobial Defense via Direct Microbial Killing and Regulating Iron Metabolism in Grass Carp (*Ctenopharyngodon idella*). Biomolecules (2020) 10(6):825. doi: 10.3390/biom10060825 PMC7356000 32481513

[36] Alvarez CA Acosta F Montero D Guzman F Torres E Vega B. Synthetic Hepcidin From Fish: Uptake and Protection Against *Vibrio Anguillarum* in Sea Bass (*Dicentrarchus Labrax*). Fish Shellfish Immunol (2016) 55:662–70. doi: 10.1016/j.fsi.2016.06.035 27368538

[37] Torrence JD Bothwell TH. Tissue Iron Stores. In: Cook JD, editor. Methods in Haematology. New York: Churchill Livingston Press (1980). p. 104–9.

[38] Xie F Xiao P Chen D Xu L Zhang B. Mirdeepfinder: A miRNA Analysis Tool for Deep Sequencing of Plant Small RNAs. Plant Mol Biol (2012) 80:75–84. doi: 10.1007/s11103-012-9885-2 22290409

[39] Morehead MS Scarbrough C. Emergence of Global Antibiotic Resistance. Prim Care (2018) 45:467–84. doi: 10.1016/j.pop.2018.05.006 30115335

[40] Aslam B Wang W Arshad MI Khurshid M Muzammil S Rasool MH. Antibiotic Resistance: A Rundown of a Global Crisis. Infect Drug Resist (2018) 11:1645–58. doi: 10.2147/IDR.S173867 PMC6188119 30349322

[41] Wang S Zeng X Yang Q Qiao S. Antimicrobial Peptides as Potential Alternatives to Antibiotics in Food Animal Industry. Int J Mol Sci (2016) 17:603. doi: 10.3390/ijms17050603 PMC4881439 27153059

[42] Baltzer SA Brown MH. Antimicrobial Peptides: Promising Alternatives to Conventional Antibiotics. J Mol Microbiol Biotechnol (2011) 20:228–35. doi: 10.1159/000331009 21894027

[43] Abdi M Mirkalantari S Amirmozafari N. Bacterial Resistance to Antimicrobial Peptides. J Pept Sci (2019) 25:e3210. doi: 10.1002/psc.3210 31637796

[44] Joo HS Fu CI Otto M. Bacterial Strategies of Resistance to Antimicrobial Peptides. Philos Trans R Soc Lond B Biol Sci (2016) 371:20150292. doi: 10.1098/rstb.2015.0292 27160595 PMC4874390

[45] Fleitas O Agbale CM Franco OL. Bacterial Resistance to Antimicrobial Peptides: An Evolving Phenomenon. Front Biosci (Landmark Ed) (2016) 21:1013–38. doi: 10.2741/4438 27100488

[46] Sahl HG Shai Y. Bacterial Resistance to Antimicrobial Peptides. Biochim Biophys Acta (2015) 1848:3019–20. doi: 10.1016/j.bbamem.2015.08.009 26342677

[47] Ramey G Deschemin JC Durel B Canonne-Hergaux F Nicolas G Vaulont S. Hepcidin Targets Ferroportin for Degradation in Hepatocytes. Haematologica (2010) 95:501–4. doi: 10.3324/haematol.2009.014399 PMC2833082 19773263

[48] Qiao B Sugianto P Fung E Del-Castillo-Rueda A Moran-Jimenez MJ Ganz T. Hepcidin-Induced Endocytosis of Ferroportin is Dependent on Ferroportin Ubiquitination. Cell Metab (2012) 15:918–24. doi: 10.1016/j.cmet.2012.03.018 PMC3372862 22682227

[49] Aschemeyer S Qiao B Stefanova D Valore EV Sek AC Ruwe TA. Structure-Function Analysis of Ferroportin Defines the Binding Site and an Alternative Mechanism of Action of Hepcidin. Blood (2018) 131:899–910. doi: 10.1182/blood-2017-05-786590 29237594 PMC5824336

[50] Chen SL Li W Meng L Sha ZX Wang ZJ Ren GC. Molecular Cloning and Expression Analysis of a Hepcidin Antimicrobial Peptide Gene From Turbot (*Scophthalmus Maximus*). Fish Shellfish Immunol (2007) 22:172–81. doi: 10.1016/j.fsi.2006.04.004 16908195

[51] Pereiro P Figueras A Novoa B. A Novel Hepcidin-Like in Turbot (*Scophthalmus Maximus L.*) Highly Expressed After Pathogen Challenge But Not After Iron Overload. Fish Shellfish Immunol (2012) 32:879–89. doi: 10.1016/j.fsi.2012.02.016 22381569

[52] Ganz T Nemeth E. Hepcidin and Iron Homeostasis. Biochim Biophys Acta (2012) 1823:1434–43. doi: 10.1016/j.bbamcr.2012.01.014 PMC4048856 22306005

[53] Arezes J Jung G Gabayan V Valore E Ruchala P Gulig PA. Hepcidin-Induced Hypoferremia Is a Critical Host Defense Mechanism Against the Siderophilic Bacterium *Vibrio Vulnificus* . Cell Host Microbe (2015) 17:47–57. doi: 10.1016/j.chom.2014.12.001 25590758 PMC4296238

[54] Kim A Fung E Parikh SG Valore EV Gabayan V Nemeth E. A Mouse Model of Anemia of Inflammation: Complex Pathogenesis With Partial Dependence on Hepcidin. Blood (2014) 123:1129–36. doi: 10.1182/blood-2013-08-521419 PMC9632791 24357728

[55] Moreira AC Neves JV Silva T Oliveira P Gomes MS Rodrigues PN. Hepcidin-(In)dependent Mechanisms of Iron Metabolism Regulation During Infection by *Listeria* and *Salmonella* . Infect Immun (2017) 85:e00353-17. doi: 10.1128/IAI.00353-17 28652306 PMC5563588

[56] Anderson GJ Frazer DM Wilkins SJ Becker EM Millard KN Murphy TL. Relationship Between Intestinal Iron-Transporter Expression, Hepatic Hepcidin Levels and the Control of Iron Absorption. Biochem Soc Trans (2002) 30:724–6. doi: 10.1042/bst0300724 12196177

[57] Frazer DM Wilkins SJ Becker EM Vulpe CD Mckie AT Trinder D. Hepcidin Expression Inversely Correlates With the Expression of Duodenal Iron Transporters and Iron Absorption in Rats. Gastroenterology (2002) 123:835–44. doi: 10.1053/gast.2002.35353 12198710

[58] Neves JV Wilson JM Kuhl H Reinhardt R Castro LF Rodrigues PN. Natural History of SLC11 Genes in Vertebrates: Tales From the Fish World. BMC Evol Biol (2011) 11:106. doi: 10.1186/1471-2148-11-106 21501491 PMC3103463

[59] Iolascon A D'apolito M Servedio V Cimmino F Piga A Camaschella C. Microcytic Anemia and Hepatic Iron Overload in a Child With Compound Heterozygous Mutations in DMT1 (Scl11a2). Blood (2006) 107:349–54. doi: 10.1182/blood-2005-06-2477 16160008

[60] Taher AT Saliba AN. Iron Overload in Thalassemia: Different Organs at Different Rates. Hematology (2017) 2017:265–71. doi: 10.1182/asheducation-2017.1.265 PMC6142532 29222265

[61] Neves JV Caldas C Wilson JM Rodrigues PN. Molecular Mechanisms of Hepcidin Regulation in Sea Bass (*Dicentrarchus Labrax*). Fish Shellfish Immunol (2011) 31:1154–61. doi: 10.1016/j.fsi.2011.10.006 22019826

[62] Liu J Sun B Yin H Liu S. Hepcidin: A Promising Therapeutic Target for Iron Disorders: A Systematic Review. Med (Baltimore) (2016) 95:e3150. doi: 10.1097/MD.0000000000003150 PMC4998755 27057839

[63] Camaschella C Nai A Silvestri L. Iron Metabolism and Iron Disorders Revisited in the Hepcidin Era. Haematologica (2020) 105:260–72. doi: 10.3324/haematol.2019.232124 PMC7012465 31949017

[64] Chen J Jiang W Xu YW Chen RY Xu Q. Sequence Analysis of Hepcidin in Barbel Steed (*Hemibarbus Labeo*): QSHLS Motif Confers Hepcidin Iron-Regulatory Activity But Limits Its Antibacterial Activity. Dev Comp Immunol (2021) 114:103845. doi: 10.1016/j.dci.2020.103845 32888968

[65] Yang CG Liu SS Sun B Wang XL Wang N Chen SL. Iron-Metabolic Function and Potential Antibacterial Role of Hepcidin and Its Correlated Genes (Ferroportin 1 and Transferrin Receptor) in Turbot (*Scophthalmus Maximus*). Fish Shellfish Immunol (2013) 34:744–55. doi: 10.1016/j.fsi.2012.11.049 23274081

[66] Shen Y Zhao Z Zhao J Chen X Cao M Wu M. Expression and Functional Analysis of Hepcidin From Mandarin Fish (*Siniperca chuatsi*). Int J Mol Sci (2019) 20(22):5602. doi: 10.3390/ijms20225602 PMC6887715 31717495

[67] Solstad T Larsen AN Seppola M Jorgensen TO. Identification, Cloning and Expression Analysis of a Hepcidin cDNA of the Atlantic Cod (*Gadus Morhua L.*). Fish Shellfish Immunol (2008) 25:298–310. doi: 10.1016/j.fsi.2008.05.013 18602479

[68] Kim YO Park EM Nam BH Kong HJ Kim WJ Lee SJ. Identification and Molecular Characterization of Two Hepcidin Genes From Black Rockfish (*Sebastes Schlegelii*). Mol Cell Biochem (2008) 315:131–6. doi: 10.1007/s11010-008-9796-3 18496731

[69] Shike H Lauth X Westerman ME Ostland VE Carlberg JM Van Olst JC. Bass Hepcidin Is a Novel Antimicrobial Peptide Induced by Bacterial Challenge. Eur J Biochem (2002) 269:2232–7. doi: 10.1046/j.1432-1033.2002.02881.x 11985602

[70] Chang WT Pan CY Rajanbabu V Cheng CW Chen JY. Tilapia (*Oreochromis Mossambicus*) Antimicrobial Peptide, Hepcidin 1-5, Shows Antitumor Activity in Cancer Cells. Peptides (2011) 32:342–52. doi: 10.1016/j.peptides.2010.11.003 21093514

[71] Chen JY Lin WJ Lin TL. A Fish Antimicrobial Peptide, Tilapia Hepcidin TH2-3, Shows Potent Antitumor Activity Against Human Fibrosarcoma Cells. Peptides (2009) 30:1636–42. doi: 10.1016/j.peptides.2009.06.009 19539000

[72] Wei X Sarath Babu V Lin L Hu Y Zhang Y Liu X. Hepcidin Protects Grass Carp (*Ctenopharyngodon Idellus*) Against *Flavobacterium Columnare* Infection via Regulating Iron Distribution and Immune Gene Expression. Fish Shellfish Immunol (2018) 75:274–83. doi: 10.1016/j.fsi.2018.02.023 29452250

[73] Huang T Gu W Wang B Zhang Y Cui L Yao Z. Identification and Expression of the Hepcidin Gene From Brown Trout (*Salmo Trutta*) and Functional Analysis of Its Synthetic Peptide. Fish Shellfish Immunol (2019) 87:243–53. doi: 10.1016/j.fsi.2019.01.020 30648626

[74] Hu X Camus AC Aono S Morrison EE Dennis J Nusbaum KE. Channel Catfish Hepcidin Expression in Infection and Anemia. Comp Immunol Microbiol Infect Dis (2007) 30:55–69. doi: 10.1016/j.cimid.2006.10.004 17126400

[75] Alvarez CA Guzman F Cardenas C Marshall SH Mercado L. Antimicrobial Activity of Trout Hepcidin. Fish Shellfish Immunol (2014) 41:93–101. doi: 10.1016/j.fsi.2014.04.013 24794583

[76] Tao Y Zhao DM Wen Y. Expression, Purification and Antibacterial Activity of the Channel Catfish Hepcidin Mature Peptide. Protein Expr Purif (2014) 94:73–8. doi: 10.1016/j.pep.2013.11.001 24269761

[77] Hirono I Hwang JY Ono Y Kurobe T Ohira T Nozaki R. Two Different Types of Hepcidins From the Japanese Flounder *Paralichthys Olivaceus* . FEBS J (2005) 272:5257–64. doi: 10.1111/j.1742-4658.2005.04922.x 16218956

[78] Qu H Chen B Peng H Wang K. Molecular Cloning, Recombinant Expression, and Antimicrobial Activity of EC-Hepcidin3, A New Four-Cysteine Hepcidin Isoform From *Epinephelus Coioides* . Biosci Biotechnol Biochem (2013) 77:103–10. doi: 10.1271/bbb.120600 23291752

[79] Lauth X Babon JJ Stannard JA Singh S Nizet V Carlberg JM. Bass Hepcidin Synthesis, Solution Structure, Antimicrobial Activities and Synergism, and In Vivo Hepatic Response to Bacterial Infections. J Biol Chem (2005) 280:9272–82. doi: 10.1074/jbc.M411154200 15546886

[80] Wang KJ Cai JJ Cai L Qu HD Yang M Zhang M. Cloning and Expression of a Hepcidin Gene From a Marine Fish (*Pseudosciaena Crocea*) and the Antimicrobial Activity of Its Synthetic Peptide. Peptides (2009) 30:638–46. doi: 10.1016/j.peptides.2008.12.014 19150638

[81] Magana M Pushpanathan M Santos AL Leanse L Fernandez M Ioannidis A. The Value of Antimicrobial Peptides in the Age of Resistance. Lancet Infect Dis (2020) 20:e216–30. doi: 10.1016/S1473-3099(20)30327-3 32653070

[82] Haney EF Hancock RE. Peptide Design for Antimicrobial and Immunomodulatory Applications. Biopolymers (2013) 100:572–83. doi: 10.1002/bip.22250 PMC3932157 23553602

[83] Li W Separovic F O'brien-Simpson NM Wade JD. Chemically Modified and Conjugated Antimicrobial Peptides Against Superbugs. Chem Soc Rev (2021) 50:4932–73. doi: 10.1039/d0cs01026j 33710195

